# Unlocking organic phosphorus and carbon in soil aggregates under long-term P and manure inputs

**DOI:** 10.1186/s12870-026-09264-5

**Published:** 2026-06-17

**Authors:** Jagdeep Singh, Aman Bhatia, Deepak Kumar Meena, Naveen Gupta, Rajeev Kumar Gupta, Graciela Dolores Avila-Quezada, Maqsood Ul Hussan, Ali Salem, Mohamed A. Mattar

**Affiliations:** 1 Department of Soil Science, Punjab Agricultural University, Ludhiana, Punjab 141004 India; 2 Department of Agronomy, School of Agriculture, Lovely Professional University, Phagwara, Punjab 144411 India; 3 Facultad de Ciencias Agrotecnológicas, Universidad Autónoma de Chihuahua, Chihuahua, 31350 México; 4 Zhejiang Provincial Key Laboratory of Agricultural Microbiomics, Institute of Biotechnology, Zhejiang University, Hangzhou, 310058 China; 5 Civil Engineering Department, Faculty of Engineering, Minia University, Minia, 61111 Egypt; 6 Structural Diagnostics and Analysis Research Group, Faculty of Engineering and Information Technology, University of Pécs, Pécs 7622, Hungary; 7 Department of Agricultural Engineering, College of Food and Agriculture Sciences, King Saud University, P.O. Box 2460, Riyadh 11451, Saudi Arabia

**Keywords:** Organic P pools, Cattle manure, Macroaggregates, Wheat grain yield, Soil pH

## Abstract

**Background:**

Phosphorus (P) availability and soil organic carbon (SOC) dynamics within soil aggregates play critical roles in regulating nutrient uptake and crop productivity. However, the interactive effects of mineral P, organic manure, and soil P status on organic P pools remain poorly understood. This study evaluated how long-term mineral P fertilization and cattle manure (CM) influence aggregate-associated P pools, SOC retention, and wheat productivity in soils with high (HGP) and medium (MDP) Olsen-P status.

**Methods:**

A long-term field experiment consisted of mineral P fertilization rates of 0, 13, 26, and 39 kg P ha^− 1^, CM alone, and integrated P + CM treatments. Soil samples were fractionated into aggregate-size classes to quantify aggregate-associated organic P fractions and SOC, while soil pH, Olsen-P, and wheat yield were also determined to assess treatment effects under contrasting soil P status.

**Results:**

Labile organic P (LBPo) increased with mineral P up to 26 kg ha^− 1^, particularly in macroaggregates (> 2.3 mm). Integrated P26 + CM further increased organic P fractions by 18% (HGP) and 23% (MDP) compared to CM alone. Similar, but to a lesser extent, increases in LBPo and SOC were observed in aggregates of 1.18–0.6 mm and 0.3–0.075 mm. Aggregate-associated SOC increased by 18.6% (HGP) and 61.5% (MDP). While P fertilization lowered soil pH, it significantly raised Olsen-P. Maximum wheat yields were achieved at 13 kg P ha^− 1^ in HGP soils and 26 kg P ha^− 1^ in MDP soils. Integrated P + CM treatments produced the highest wheat yields under both Olsen-P status soils. In HGP soils, P13 + CM and P26 + CM resulted in statistically comparable yields, whereas P26 + CM produced the maximum yield in MDP soils.

**Conclusions:**

Soil Olsen-P status and aggregate size regulate organic P stabilization and SOC retention. Integrating CM with mineral P is the most effective strategy for enhancing aggregate-associated SOC and sustaining wheat productivity in soils with contrasting P status.

**Supplementary Information:**

The online version contains supplementary material available at 10.1186/s12870-026-09264-5.

## Introduction

Phosphorus (P) is an indispensable nutrient for crop production and, after nitrogen (N), is the second-most widely applied fertilizer worldwide [1]. As a high-nutrient-demanding crop, wheat (*Triticum aestivum* L.) serves as a primary driver of global P consumption. Currently, wheat is a cornerstone of food security, with an annual production exceeding 840 million tonnes from approximately 225 million hectares worldwide [2]. In India, it ranks as the second most important cereal crop, contributing nearly 118 million tonnes annually to the national grain basket [3]. To sustain these high productivity levels, intensive fertilization, particularly of P, is required; however, the efficacy of these inputs often depends on the soil’s existing physicochemical state and biological health [4, 5]. Despite its importance, the efficiency of applied P is very low, with only 10–25% taken up by plants [5], while the remainder is immobilized through interactions with Fe^3+^ and Al^3+^ in acidic soils, with Ca^2+^ in alkaline soils, or converted into less labile fractions [6, 7]. To compensate for these losses, farmers often apply amounts well above crop requirements [8, 9], leading to a gradual build-up of residual or “legacy” P in agricultural soils. This has resulted in a wide range of Olsen-P levels in intensively managed fields, with consequences for nutrient cycling, soil fertility, and environmental sustainability.

In addition to enriching soil P pools, long-term fertilizer P use affects soil properties by influencing the turnover of soil organic carbon (SOC) and aggregate stability [7]. Inputs of crop residues and organic matter, together with Fe and Al oxides, promote aggregation [10]. Soil aggregates, in turn, are fundamental to mediating SOC and P stabilization, serving as microsites that protect nutrients from rapid turnover and loss [1, 11]. Recent research has shown that the co-application of organic amendments with mineral fertilizers significantly enhances soil health [12, 13]. Yet the effects of P fertilization on aggregate-associated P fractions remain unclear, with contrasting evidence across studies. Some reports highlight enrichment of added P in microaggregates (< 0.25 mm) and fine fractions due to their high surface area and reactivity [14, 15], whereas others show stronger P retention in macroaggregates (0.25–1 mm) because of their organic matter content and greater C–P linkages [13]. Organic inputs, particularly cattle manure (CM), which is readily available in mixed farming systems, have also been shown to alter P distribution among aggregate fractions [16]. Variations in soil C and P fractions are intrinsically linked to microbial biomass and hyphosphere fungi–bacteria–plant interactions, which regulate P trade-offs within the soil-plant system [6]. However, contrasting results across sites suggest that P stabilization within aggregates is not uniform but depends strongly on soil fertility status, organic inputs, and management history [17, 18].

Although organic P accounts for 30–65% of total soil P [19], much research at the aggregate scale has focused on inorganic fertilizer inputs [20]. Organic P in soil exists in several fractions, including labile forms such as microbial biomass P and easily mineralizable monoester P, moderately labile forms including phytate-associated inositol phosphates, and recalcitrant nonlabile forms that are strongly adsorbed to mineral surfaces or occluded within aggregates [21]. Recent work has highlighted that the relative distribution of these fractions is governed not only by P input levels but also by SOC content, microbial community composition, and aggregate architecture [11]. Long-term, integrated application of mineral P with organic amendments, such as CM, has increasingly been recognized as a strategy that both increases labile organic P pools and promotes SOC accumulation by stimulating microbial biomass and phosphatase activity [22]. Specifically, the combined P + CM approach has been shown to shift P from recalcitrant, nonlabile pools toward more plant-available, labile, and moderately labile fractions, with effects varying across aggregate size classes [23]. Additionally, manure-derived inputs contribute directly to organic P via partially mineralized organic compounds that interact with soil minerals within aggregates, influencing P stabilization pathways [24]. However, most existing studies on aggregate-associated organic P are conducted under uniform soil fertility, leaving the responses of organic P fractions to different Olsen-P levels poorly understood. The transformation and stabilization of organic P within aggregates, particularly across soils with varying levels of legacy P accumulation, remain incompletely understood. Addressing these gaps is crucial for clarifying aggregate-associated P sources and sinks, improving fertilizer-use efficiency, and supporting the development of sustainable nutrient-management strategies in high-input cropping systems.

The maize-wheat rotation in Northwest India, within the Indo-Gangetic Plains, represents one of the world’s most intensively cultivated systems [25]. Prolonged reliance on heavy P applications has led to considerable buildup of variable levels of unused soil residual P in many cultivated fields, making the region suitable for examining how P fertilization at different Olsen-P levels affects aggregate-associated organic P pools and SOC retention within aggregate fractions. Accordingly, the present study utilized an ongoing long-term field experiment (started in 2011) to examine the effects of mineral P fertilization and CM application on aggregate-associated organic P pools and SOC dynamics in soils differing in Olsen-P status under the maize-wheat system. While previous studies have investigated links between long-term P and manure inputs and soil P dynamics or SOC in aggregates, the present study integrates organic P fractionation across aggregate size classes under contrasting Olsen-P fertility gradients established through a controlled long-term experiment, thereby providing aggregate-scale mechanistic insights under defined soil P gradient conditions that have not been simultaneously examined in prior work.

## Materials and methods

### Site description

A long-term experiment in the maize-wheat cropping system was established in 2011 at Punjab Agricultural University’s Department of Soil Science in Ludhiana, India (30°54’ N, 75°47’ E). As part of this study, soil and plant samples were collected and analyzed after the 2023 wheat harvest. The experimental site has a subtropical, semi-arid climate, and the soil is loamy sand.

### Experimental detail and crop management

The field experiment was initiated in 2011, following the establishment of three simulated P-gradient strips in 2010 through a single application of P fertilizer. Langmuir P sorption isotherms were used to calculate the P fertilizer dose required to achieve the desired Olsen-P levels [26]. Multiple wetting-drying cycles, followed by plowing with a disc harrow, were performed to naturally stabilize soil P levels. In the present context, two of the three P fertility strips were selected for detailed investigation. These included the high P (HGP) strip, characterized by Olsen-P levels exceeding 22.3 mg kg^− 1^, and the medium P (MDP) strip, with Olsen-P levels ranging from 5.6 to 10.0 mg kg^− 1^. At the commencement of the experiment, composite soil samples collected from the 0–0.15 m layer exhibited the following properties: pH 7.6, electrical conductivity 0.23 dS m^− 1^, SOC 2.91 g kg^− 1^, KMnO_4_-N 42.9 mg kg^− 1^, 1 M NH_4_OAc-K 38.7 mg kg^− 1^, and Olsen-P 8.52 and 25.1 mg kg^− 1^ in the MDP and HGP strips, respectively. However, in 2023, the physicochemical properties of soil samples ranged from pH 6.56–7.46, electrical conductivity 0.22–0.25 dS m^− 1^, SOC 2.30–3.34 g kg^− 1^, KMnO_4_-N 38.4–58.1 mg kg^− 1^, and 1 M NH_4_OAc-K 41.0-43.5 mg kg^− 1^ across soils with different Olsen-P status. Olsen-P varied from 4.8 to 16.9 and 10.4–35.5 mg P kg^− 1^ in the MDP and HGP strips, respectively.

The experiment comprised P application rates (0, 13, 26, 39 kg ha^− 1^) and CM applied at 15 t ha^− 1^ (50% moisture content), containing 0.82% N, 0.12% P, and 0.82% K. Based on these, seven treatment combinations were evaluated: P0, P13, P26, P39, CM, P13CM, and P26CM. The treatments were arranged in a randomized block design with three replicates per strip, with each plot measuring 8 × 3 m. A recommended dose of N at 120 kg N ha^− 1^ was added through urea, and K at 25 kg ha^− 1^ using KCl was applied to each wheat and maize. P, as single superphosphate (SSP), and K were incorporated at sowing time. N was administered in wheat, with 20 kg ha^− 1^ at sowing and the remaining half in two equal splits after the 1st and 2nd irrigations. Well-decomposed fresh CM was incorporated only once per annual cropping cycle, one day before maize sowing, and the effects observed in the subsequent wheat crop represent residual effects of CM application. Wheat was planted in early November and harvested in April at maturity. The first irrigation was applied three weeks after sowing, with additional irrigations as per crop need. The plots were maintained weed-free and protected from insects, pests, and diseases using chemicals in accordance with regional standard crop management practices.

### Soil and aggregate sampling

Surface soil samples (0–0.15 m) were collected from ‘MDP’ and ‘HGP’ Olsen-P status soils after the wheat harvest in 2023, following 13 years of continuous treatment application. Soil samples were thoroughly mixed, air-dried, and then sieved through a 2-mm mesh for subsequent analysis. However, soil clods (0–0.15 m) were carefully collected in an undisturbed state using a shovel after the wheat crop, following the “wet sieving method” [27]. Air-dried soil aggregates of size 4 mm were subjected to mechanical agitation in Yoder’s apparatus for 30 min to fractionate aggregates into different size fractions of 2.30 mm, 1.18 mm, 0.60 mm, 0.30 mm, and 0.075 mm. After wet sieving, the five recovered fractions were pooled by mass into three operational aggregate-size classes prior to chemical analysis: large macroaggregates (> 2.3 mm), small macroaggregates (1.18–0.6 mm; the 1.18 mm and 0.60 mm fractions combined in proportion to their recovered mass), and microaggregates plus silt-clay (0.3–0.075 mm; the 0.30 mm and 0.075 mm fractions combined in proportion to their recovered mass). Pooling was necessary to ensure sufficient sample mass for the multi-step sequential organic P extraction. All subsequent organic P and SOC determinations refer to these three pooled classes.

### Soil properties, aggregate assessment, and organic phosphorus fractionation

Soil pH was measured with a pH meter following Jackson [28]. SOC was estimated using the Walkley and Black [29] wet combustion technique with rapid titration. Olsen-P was extracted using the method of Olsen et al. [30].

**Fig. 1:**
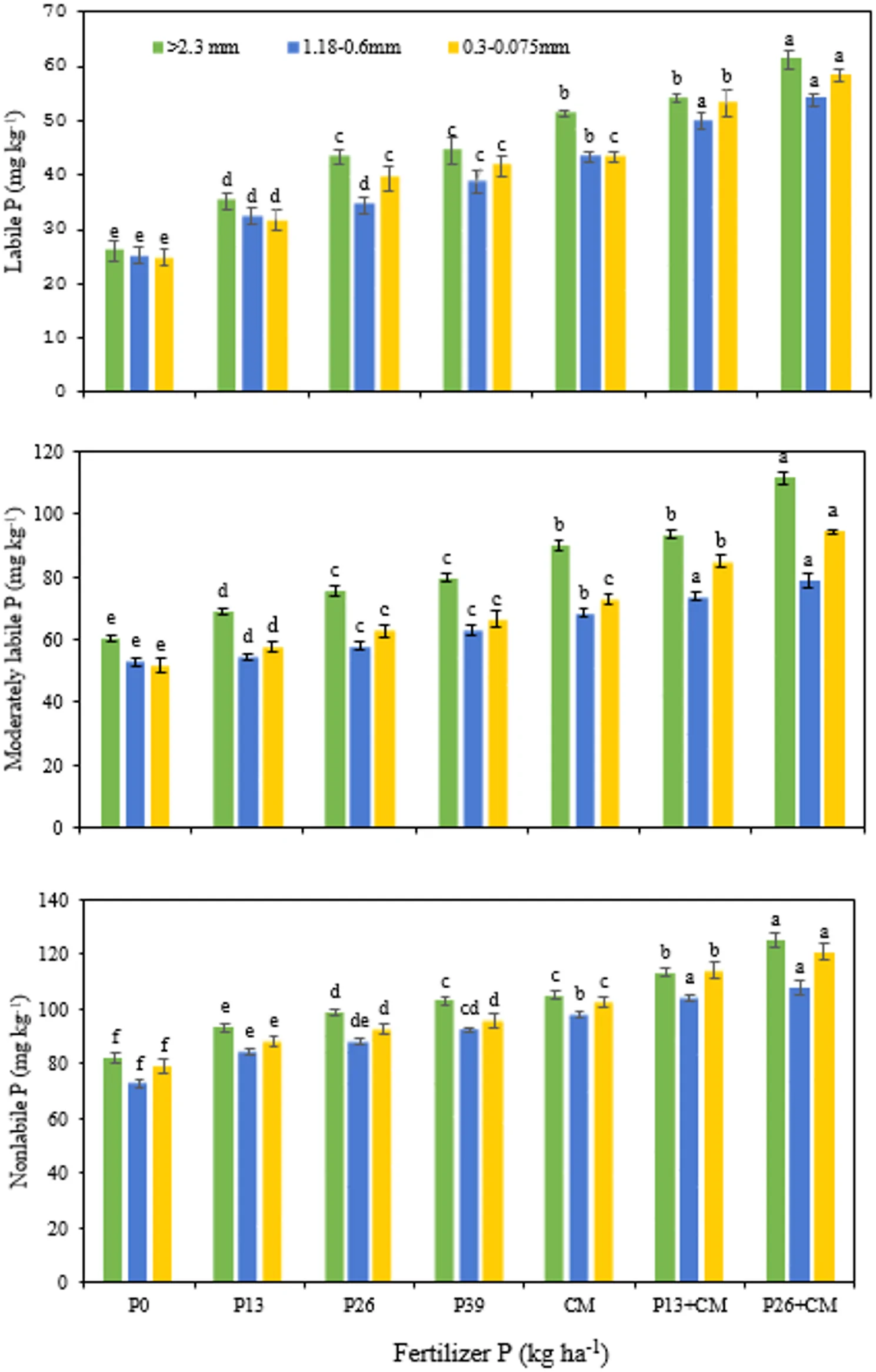
Labile P, moderately labile P, and non-labile P as affected by P application in high P soils under different aggregate size classes. Mean values with different letters indicate significant differences (*p* < 0.05) between treatments by Duncan’s multiple range test. *Vertical lines* show standard error of the mean

Soil organic P fractions, viz., labile organic P (LBPo), moderately labile P (MLP_O_), and nonlabile P (NLP_O_), were extracted from different aggregate size classes (> 2.3 mm, 1.18–0.6 mm, and 0.3–0.075 mm) and determined using the method developed by Kovar and Pierzynski [31], as shown in Fig. 1S.

### Statistical assessment

Data were analyzed using SPSS Version 23.0. The experiment was analyzed using a randomized block design with two fixed factors: nutrient management treatment (seven levels: P0, P13, P26, P39, CM, P13CM, and P26CM) and soil Olsen-P status (MDP and HGP). Replications were treated as random blocks nested within Olsen-P status. The Treatment × Olsen-P status interaction was tested. Where ANOVA indicated significant effects (*p* < 0.05), treatment means were separated using Duncan’s Multiple Range Test. Pearson’s correlation analysis was performed using Origin 2025 software.

## Results

### P fertilization and CM influence aggregate-associated organic P fractions

**Fig. 2:**
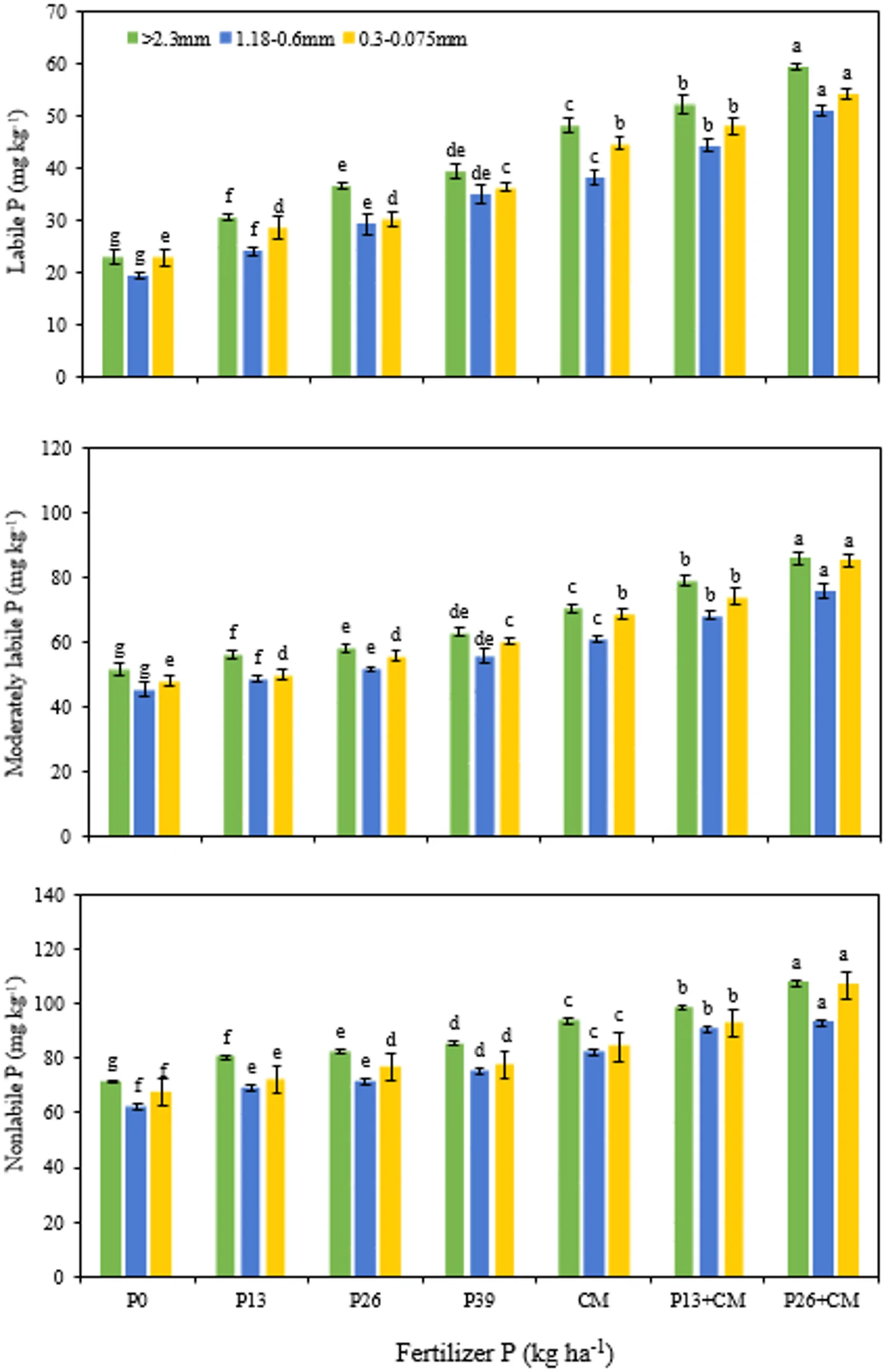
Labile P, moderately labile P, and non-labile P as affected by P application in medium P soils under different aggregate size classes. Mean values with different letters indicate significant differences (*p* < 0.05) between treatments by Duncan’s multiple range test. *Vertical lines* show standard error of the mean

Long-term P fertilization had a considerable impact on all organic P fractions (LBPo, MLP_O_, and NLP_O_) across all aggregate size classes (> 2.3 mm, 1.18–0.6 mm, and 0.3–0.075 mm) in both Olsen-P status soils (Figs. 1 and 2). The LBP_O_ associated with the > 2.3 mm aggregate size class increased significantly with increasing P fertilization rates in both MDP and HGP soils. The LBP_O_ increased by up to 65% in HGP and 60% in MDP soils with the application of 26 kg P ha^− 1^, compared with no P application. Increasing P rates up to 39 kg P ha^− 1^ had no significant effect on labile P in either P-status soil. The combined application of P and CM further increased the LBP_O_ fraction, with the highest increases of 18% in HGP and 23% in MDP soils in the P26 + CM treatment compared with CM alone. A similar increasing trend in LBPo was observed in the 1.18–0.6 mm and 0.3–0.075 mm aggregate size classes, though values were lower than those in the > 2.3 mm class. Correspondingly, MLP_O_ also increased with increasing P addition rates. However, the overall concentration of MLP_O_ was higher than that of LBP_O_, ranging from 51 to 111 mg kg^− 1^ and 45–85 mg kg^− 1^ in HGP and MDP soils, respectively. MLP_O_ associated with the > 2.3 mm aggregate size class increased by 24% and 12% in HGP and MDP soils, respectively, with the application of 26 kg P ha^− 1^ among fertilizer P treatments. Similar to LBPo, the highest increase in MLP_O_ concentration was observed in the P26 + CM treatment, accounting for increases of 24% in HGP and 21% in MDP soils compared with CM alone. An increasing trend with increasing levels of P fertilizer addition and integrated use of P and CM was also observed in the 1.18–0.6 mm and 0.3–0.075 mm aggregate size classes. NLP_O_ was the major organic P fraction, ranging from 72 to 125 mg kg^− 1^ in HGP and 62–107 mg kg^− 1^ in MDP soils. Among the aggregate size classes, > 2.3 mm exhibited the highest NLP_O_ accumulation with fertilizer P addition, showing increases of 25% in HGP and 19% in MDP in the P39 treatment compared with no P addition. The P26 + CM treatment exhibited the highest increases of 18% and 14% in HGP and MDP soils, respectively, compared with CM alone.

### Effect of P fertilization and CM application on aggregate-associated SOC

P fertilization had differential effects on SOC across aggregate size classes under varying Olsen-P soil status (Table 1). In HGP and MDP soils, fertilizer P application significantly increased SOC in the > 2.3 mm aggregate-size class as compared to no P fertilization. In HGP soils, no significant response was observed above 13 kg P ha^− 1^; however, a significant response was observed up to 26 kg P ha^− 1^ in MDP soils. Compared with CM alone, P26 + CM increased SOC in the > 2.3 mm fraction from 1.18 ± 0.15 to 1.40 ± 0.56 g kg^− 1^ in HGP soils and from 0.52 ± 0.10 to 0.84 ± 0.20 g kg^− 1^ in MDP soils (Table 1). Likewise, SOC in the 1.18–0.6 mm and 0.3–0.075 mm aggregate size classes also showed a comparable increasing trend to that observed in the > 2.3 mm aggregate size class. Overall, HGP soils exhibited higher aggregate-associated SOC across all aggregate size classes.

**Table 1:** Effect of added P and CM on aggregate-associated soil organic carbon in different aggregate size classes in high P and medium P soils

Fertilizer *P*	Aggregate associated soil organic carbon (g kg^-1^)					
	High *P*			Medium *P*		
	>2.3 mm	1.18–0.6 mm	0.3–0.075 mm	>2.3 mm	1.18–0.6 mm	0.3–0.075 mm
P0	0.45±0.30^d^	0.23±0.25^e^	0.30±0.06^d^	0.20±0.06^g^	0.31±0.05^e^	0.26±0.20^d^
P13	0.87 ±0.06^c^	0.32±0.31^cd^	0.56±0.06^c^	0.30±0.16^f^	0.40±0.04^d^	0.33±0.30^c^
P26	0.81±0.05^c^	0.46±0.29^cd^	0.63±0.07^bc^	0.39±0.15^e^	0.46±0.12^cd^	0.35±0.13^c^
P39	0.93±0.20^c^	0.56±0.11^bc^	0.69±0.17^b^	0.42±0.06^de^	0.49±0.09^cd^	0.35±0.19^c^
CM	1.18±0.15^b^	0.81±0.19^b^	0.96±0.03^a^	0.52±0.10^c^	0.62±0.02^ab^	0.46±0.27^bc^
P13+CM	1.30±0.33^ab^	0.85±0.13^ab^	0.99±0.14^a^	0.65±0.13^b^	0.67±0.08^a^	0.59±0.04^ab^
P26+CM	1.40±0.56^a^	1.16±0.25^a^	1.01±0.08^a^	0.84±0.20^a^	0.72±0.11^a^	0.73±0.12^ab^

Means within a column followed by different letters are significantly different at *p*< 0.05 by Duncan’s multiple range test. Values represent means ± standard error (SE) of three replications

### P and CM effects on soil pH, Olsen-P, and SOC

Continuous fertilizer P application decreased soil pH in both HGP and MDP soils (Table 2). Among the fertilizer P treatments, 39 kg P ha^− 1^ produced the lowest pH in HGP soils (7.12 ± 0.06), compared with P0 (7.30 ± 0.16) though this reduction was not statistically different from P26 (7.14 ± 0.12). In MDP soils, a significantly higher decline in pH was recorded with 26 and 39 kg P ha^− 1^, both of which led to greater acidification than the lower P rates. In HGP soils, the combined application of CM and P produced the most significant pH decrease under the P26 + CM treatment, whereas no such effect was evident in MDP soils. In contrast to pH, Olsen-P increased significantly with increasing fertilizer P rates in both P-status soils. The highest increases were 164% in HGP and 254% in MDP soils with the addition of 39 kg P ha^− 1^ over no-P application. Significantly higher Olsen-P was also observed in the P13 + CM and P26 + CM treatments (Table 2), with the maximum increase recorded in the P26 + CM treatment, regardless of soil P status. Moreover, SOC content increased with P addition rates up to a certain level. The SOC increased from 3.1 ± 0.04 to 3.5 ± 0.18 g kg^− 1^ in HGP soils and from 2.1 ± 0.06 to 3.1 ± 0.03 g kg^− 1^ in MDP soils with P26 application compared with P0 (Table 2). Additionally, the combined application of P and CM significantly increased SOC content, with the largest increase observed in the P26 + CM treatment, which was not statistically different from P13 + CM in MDP soils.

**Table 2:** Effect of added P and CM on soil chemical properties in high P and medium P soils

Treatment	High *P*			Medium *P*		
	pH	Olsen-*P* (mg kg^-1^)	SOC (g kg^-1^)	pH	Olsen-*P* (mg kg^-1^)	SOC (g kg^-1^)
**P0**	7.30±0.16^a^	10.4 ±1.58^f^	3.1±0.04^d^	7.49±0.05^a^	4.8±1.12^f^	2.1±0.06^b^
**P13**	7.21±0.13^b^	17.1±2.45 ^e^	3.2±0.03^d^	7.34±0.03^b^	6.6±1.43^e^	3.0±0.05^a^
**P26**	7.14±0.12^bc^	32.0 ± 1.69^c^	3.5±0.18^c^	7.28±0.15^cd^	14.8±2.18^c^	3.1±0.03^a^
**P39**	7.12±0.06^bc^	35.5 ±1.97^a^	3.6±0.04^c^	7.26±0.18^cd^	16.9±1.73^a^	3.2±0.010^a^
**CM**	7.18±0.19^b^	10.7 ±2.41^f^	3.2±0.04^d^	7.29±0.16^c^	5.0±1.01^f^	2.4±0.10^b^
**P13+CM**	7.15±0.12^b^	18.9 ±1.76^d^	3.9±0.05^b^	7.25±0.08^c^	7.6±1.63^d^	2.9±0.05^a^
**P26+CM**	7.08±0.26^c^	34.1 ±1.56^b^	4.4±0.03^a^	7.24±0.02^c^	15.7±2.21^b^	3.2±0.05^a^

Means within a column followed by different letters are significantly different at *p*< 0.05 by Duncan’s multiple range test, *SOC* Soil organic carbon

Values represent means ± standard error (SE) of three replication

### Crop yield as influenced by P and CM

The mean wheat grain yield increased significantly with P application up to 13 kg P ha^− 1^ in HGP and up to 26 kg P ha^− 1^ in MDP (Table 3). Beyond these rates, no further yield response was observed. Moreover, the combined application of P with CM significantly increased grain yield in the P26 + CM treatment compared with P26 alone in MDP soils, whereas in HGP soils, P13 + CM produced a yield comparable to P26 + CM. The comparison of mean wheat yields between the two Olsen-P status soils revealed a significant 8.7% higher grain yield in HGP soils than in MDP soils.

**Table 3:** Average wheat grain yield (t ha^-1^) as affected by P fertilization in high P and medium P soils (pooled mean of 13 years)

Treatments	High *P*	Medium *P*	Mean
P0	4.03^f^	3.11^h^	3.57^e^
P13	4.93^c^	4.26^e^	4.60^c^
P26	5.08^b^	5.00^c^	5.04^b^
P39	5.16^b^	5.05^b^	5.11^b^
CM	4.62^d^	3.82^g^	4.22^d^
P13+CM	5.34^a^	5.21^b^	5.28^a^
P26+CM	5.38^a^	5.31^a^	5.34^a^
Mean	4.93^a^	4.53^b^	
LSD (5%)	*P* = 0.12, *Olsen-P*=0.06,* P × Olsen-P* =0.16		

Means in the ‘Mean’ column and ‘Mean’ row indicate significant effects of P fertilization and Olsen-P status, respectively. Different lowercase letters denote significant differences among treatment combinations for the P × Olsen-P interaction

### Correlation matrix in HGP and MDP soils

**Fig. 3:**
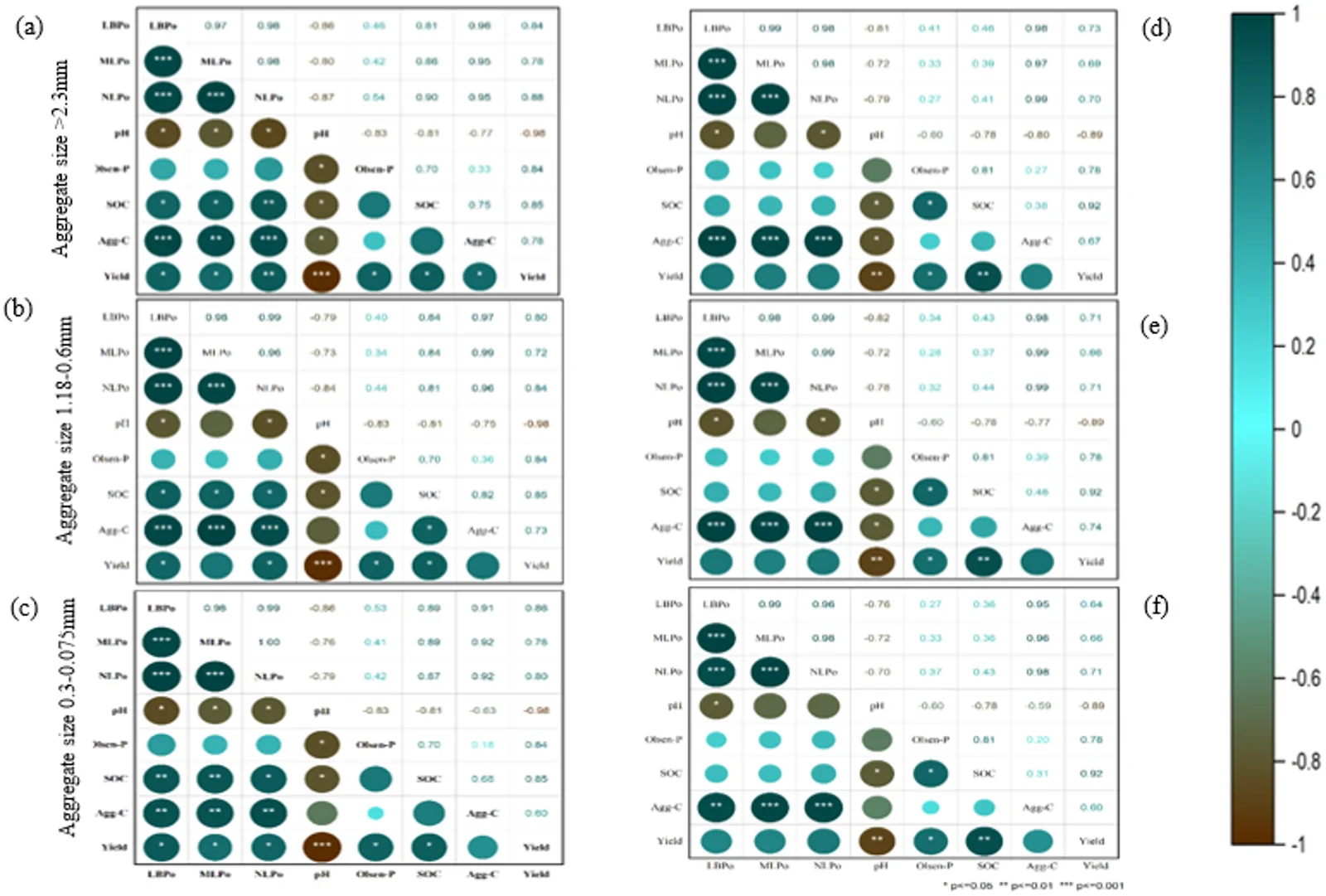
Heatmap of correlations among aggregate-associated soil organic P fractions (LBPo-labile P; MLPo- moderately labile P; NLPo- nonlabile P), pH, Olsen-P, soil organic carbon (SOC), aggregate-associated SOC, and crop yield across different aggregate size classes in high P (**a**, **b**, **c**) and medium P (**d**, **e**, **f**) soils

The correlation heatmaps showed strong positive relationships among organic P fractions, SOC, aggregate-associated SOC, and crop yield in HGP soils (Fig. 3a-c). These strong correlations were most evident in the > 2.3 mm aggregates. Soil pH showed a strong negative correlation with all these variables. Similar trends appeared in the 1.18–0.6 mm and 0.3–0.075 mm aggregates, but with slightly lower magnitude (Fig. 3b, c). In MDP soils, the positive correlations among P fractions, SOC pools, and yield were weaker (Fig. 3d-f). The negative correlation with soil pH was also less pronounced. Overall, higher P availability was associated with stronger covariation among organic P fractions, aggregate-associated SOC, and crop yield.

## Discussion

### Aggregate-associated organic P fractions as influenced by P inputs and Olsen-P status

Aggregate-associated organic P fractions were strongly influenced by P application and Olsen-P status. The increased LBPo availability at 26 kg P ha^− 1^ in aggregates > 2.3 mm in both HGP and MDP soils may be due to enhanced microbial activity in larger pore spaces, which facilitates organic P mineralization [32]. This interpretation is consistent with evidence that variations in soil C and P fractions are closely associated with microbial biomass dynamics, which regulate organic P turnover within aggregates [1]. Beyond 26 kg P ha^− 1^, continuous application did not further increase organic P levels, as sorption capacity and microbial immobilization became saturated, leaving excess P in inorganic forms [33]. The combined application of P and CM further enhanced LBPo (18% in HGP and 23% in MDP soils in P26 + CM over CM alone), most likely reflecting manure-derived organic inputs and simulated microbial biomass associated with macroaggregate formation [34]. In contrast, LBPo concentrations were lower in smaller aggregates (1.18–0.6 mm and 0.3–0.075 mm) than in aggregates > 2.3 mm, due to their compact structure, which negatively affects root development and reduces roots’ capacity to absorb water and nutrients. The considerable increase in MLPo within soil aggregates resulting from long-term P and CM addition can be attributed to key alterations in soil properties, including reduced pH and elevated SOC levels, which improve P solubility and play both direct and indirect roles in the cycling of soil P [35, 36]. Microbial decomposition of organic amendments further accelerates organic matter turnover and the associated release of P. Moreover, the greater accumulation of MLPo in soil aggregates > 2.3 mm than in smaller ones likely reflects SOC-induced aggregation, which stabilizes the structure of larger aggregates and mitigates the dispersal of finer mineral particles [5, 32]. Also, the increased MLPo concentration in HGP soils can be ascribed to higher native P reserves resulting from long-term P fertilization. The NLPo, inherently insoluble and stable, remains largely inaccessible to plants due to its strong association with mineral surfaces [37]. The progressive accumulation of NLPo with increasing fertilizer P addition may be attributed to adsorption onto mineral surfaces, thereby sequestering it in a stable form [38]. Higher retention of NLPo was found in HGP soils relative to MDP soils, owing to greater P accumulation, as evident from the Olsen-P status given in Table 2. Higher NLPo concentrations recorded during elevated P addition rates were likely due to P saturation from excessive P fertilization. Over time, a portion of this accumulated non-labile P may slowly transform into labile and moderately labile pools, thereby enhancing its potential availability to plants [6, 34]; however, continued excessive fertilizer inputs could increase the risk of environmental P mobilization and off-site transport.

### Soil Olsen-P status associated with aggregate-scale SOC dynamics

The differential responses of aggregate-associated SOC to P application in both MDP and HGP soils can be attributed to their differing Olsen-P levels. Du et al. [39] reported an increasing trend in SOC with aggregate size, which they linked to greater aggregate stability under P fertilization. Fink et al. [40] reported that when P competes with SOC for binding sites on soil particles and minerals, SOC bioavailability to microbes increases, altering SOC distribution within aggregates and aggregate stability. In high-soil-P environments, diminishing returns in SOC beyond lower P rates of 13 kg P ha^− 1^ were primarily due to P saturation; once a threshold P level is attained, the soil no longer serves as a C sink [41]. Changes in soil C: P ratios under high P supply can further stimulate microbial growth and accelerate decomposition of labile carbon pools [42]. Zhang et al. [43] reported that P application in high Olsen-P soils enhanced microbial activity, thereby enabling microbes to use carbon stored in larger soil aggregates. Moreover, higher P rates affect C: P ratios in soil, stimulating microbial growth and promoting the decomposition of labile organic matter fractions that would otherwise be resistant to microbial degradation, thereby decreasing the formation and stabilization of macroaggregates and reducing SOC in macroaggregates [44]. Conversely, the increase in aggregate-associated SOC with P application in MDP soils up to 26 kg P ha^− 1^ aligns with earlier studies suggesting that moderate P addition enhances SOC stabilization within aggregates by improving aggregate stability and reducing SOC mineralization [45, 46]. Furthermore, the greatest increase in aggregate-associated SOC with the combined application of P and CM may be attributed to bioproducts generated during CM decomposition and to binding contributions from root exudates and fungal hyphae, as documented in similar systems [47]. Similar improvements in active carbon fractions and maize productivity following organic amendment application have been reported in organic-matter-deficient soils [4]. Notably, the greater SOC accumulation observed in aggregates > 2.3 mm, compared with the smaller size classes (1.18–0.6 mm and 0.3–0.075 mm), across soils with contrasting Olsen-P levels, could be due to the development and stabilization of soil macroaggregates. This process involves the binding of free primary particles by persistent binding agents, facilitating the formation of macroaggregates [48]. Thus, the variability in SOC responses between HGP and MDP soils suggests that soil P status is a critical determinant of the efficacy of P fertilization.

### Soil chemical dynamics under long-term P and CM application

The decline in soil pH observed with long-term P application may reflect the combined influence of SSP chemistry, soil buffering capacity, nutrient uptake and removal, and interactions between mineral fertilizers and organic amendments. Similar trends have been reported under prolonged P fertilization in comparable agroecosystems [49]. Touhami et al. [50] reported that the application of SSP introduces phosphate ions (H_2_PO_4_⁻) into the soil solution, which react with water to form phosphoric acid (H_3_PO_4_), which dissociates to release hydrogen ions (H⁺), thereby reducing soil pH. In MDP soils, the stronger decline in pH at higher P rates suggests that acidification pressure exceeded the soil’s buffering capacity. In contrast, the increase in soil pH following CM application alone may be attributed to its higher organic matter content and buffering capacity [51], which promote soil structural development and improve cation exchange capacity (CEC) [52]. Compared with the combined P26 + CM treatment, CM alone maintained relatively higher pH values, which may reflect the buffering influence of organic amendments reported in previous studies [53, 54]. The increased CEC enables the soil to retain more basic cations, which buffer against pH changes and slow down soil acidification [55]. The substantial increase in Olsen-P at higher P rates is explained by saturation of sorption sites on Fe/Al oxides and clay minerals, thereby increasing P concentrations in the soil solution [56]. The synergistic effect of P + CM (particularly P26 + CM) arises from enhanced SOC content and soil structure, which reduce P fixation and mobilize both native and applied P [57]. Organic inputs have been reported to stimulate microbial activity and the release of phosphatases that can mobilize organically bound P [58]. The SOC levels rose with increasing P up to P26, then plateaued at higher P. This suggests that once P supply is adequate, other factors, such as N availability or microbial turnover, limit further SOC accumulation. The significant SOC gains with P + CM reflect the addition of lignin-rich organic matter and microbial by-products from manure decomposition, which promote macroaggregate formation and stabilize SOC in larger size classes [59]. These findings indicate that while high SSP rates can acidify soils, the integrated use of P fertilizer with CM enhances soil fertility by simultaneously improving pH buffering, Olsen-P availability, and SOC accumulation [60].

### Crop yield and its coupling with organic P fractions and SOC stabilization

The increased wheat yields observed with 13 kg P ha^− 1^ in HGP and 26 kg P ha^− 1^ in MDP soils were attributed to a higher proportion of macroaggregates at these P rates, which support improved root growth, and to a positive P balance, indicating enhanced P availability, as reported in our earlier study [61, 62]. These outcomes align with Li et al. [63], who reported that sustained P fertilization improves soil aggregation and increases P availability, thereby supporting greater plant uptake and resulting in higher crop productivity [64]. Furthermore, improved crop yield from the combined use of P and CM may result from improved soil properties and an adequate nutrient supply. Many researchers have reported improved crop yields with inorganic and organic fertilizers [65, 66].

The strong positive correlation among organic P fractions across aggregate size classes highlights the interconnectedness of soil P pools. Similar findings were reported by Li et al. [63], who observed a comparable increase in organic P fractions, reinforcing dynamic interactions within soil P pools. Furthermore, the positive association between organic P fractions and aggregate-associated SOC, reflected in higher R^2^ values (Fig. 3a, b), suggests that increased SOC concentrations in soil aggregates of varying sizes play a pivotal role in stabilizing and retaining organic P fractions within aggregates. Such covariation has been reported elsewhere to be linked to microbial biomass-mediated transformations and to the soil C: P stoichiometric balance [1, 5]. The negative relationship between soil pH and organic P fractions may reflect increased sorption of soluble organic P as pH decreases [6, 58]. Kataria and Singh [67] reported that changes in pH can influence organic P forms and phosphatase enzyme activity, although the exact mechanisms remain poorly understood.

## Conclusion

The > 2.3 mm aggregate fraction (macroaggregates) accumulated the highest levels of labile P fractions, regardless of soil Olsen-P status. CM incorporation, together with P application, had a synergistic effect, increasing labile and non-labile organic P forms. Specifically, the integrated P26 + CM treatment enhanced labile organic P by up to 23% in MDP soils and 18% in HDP soils compared with CM alone, demonstrating a strong interactive effect of mineral and organic inputs. P fertilization increased SOC concentrations across all aggregate size classes. However, the greatest relative increase in aggregate-associated SOC with P application was observed in MDP soils. Long-term P application across soils with varying Olsen-P status affects soil pH, Olsen-P, and SOC, indicating that the strength of nutrient interactions is modulated by initial Olsen-P status. Under the conditions of a long-term experiment, wheat yield in HGP soils was maintained at 13 kg P ha^− 1^, suggesting the potential for reduced fertilizer P inputs relative to MDP soils. However, these findings should be interpreted within the context of the present study and demand further validation across different environments and management scenarios. These findings support efficient P management decisions under intensive cropping systems. Future research should explore diverse fertilization strategies, organic amendments, and microbial mechanisms that drive P-SOC interactions across soil types to enhance sustainable P cycling.

## Supplementary Information

Supplementary Material 1.

## Data Availability

The datasets used and/or analyzed during the current study are available from the corresponding author upon reasonable request.
