# Supplementary material for: Unlocking organic phosphorus and carbon in soil aggregates under long-term P and manure inputs
Source: BMC Plant Biol. 2026 Jun 17;26:1457. doi: 10.1186/s12870-026-09264-5 (PMC13508231; doi:10.1186/s12870-026-09264-5)
Supplement: Supplementary file 1 [file 12870_2026_9264_MOESM1_ESM.docx]

Labile Pi

Persulfate digestion

**Residue**

25 ml of 0.5M NaHCO_3_, shake for 16 hrs, centrifuge, and filter

Aliquot

Total Labile P

**Labile P (LBPo) =Total Labile P- Labile Pi**

Moderately Labile Pi

**Residue**

Aliquot

25 ml of 1.0M HCl, shake for 3 hrs, centrifuge, and filter

Aliquot

Persulfate digestion

Total P

**Moderately Labile P (MLPo) =Total P- Pi**

Rinse with deionized water for 5 minutes, centrifuge

Dissolve in 25 ml 1.0 M H_2_SO_4_, shake 24 h

Ash at 550^o^C, 1 h

Nonlabile P (NLPo)

Discard Supernatant

**Residue**

**Figure 1S.** Schematic representation of the procedure for determining organic phosphorus fractionation in soil.
